# Artificial intelligence (AI) for paediatric fracture detection: a multireader multicase (MRMC) study protocol

**DOI:** 10.1136/bmjopen-2024-084448

**Published:** 2024-12-07

**Authors:** Susan C Shelmerdine, Cato Pauling, Emma Allan, Dean Langan, Emily Ashworth, Ka-Wai Yung, Joy Barber, Saira Haque, David Rosewarne, Nick Woznitza, Sarim Ather, Alex Novak, Kanthan Theivendran, Owen J Arthurs

**Affiliations:** 1 Clinical Radiology, Great Ormond Street Hospital for Children, London, UK; 2 UCL Great Ormond Street Institute of Child Health, London, UK; 3 Great Ormond Street Hospital NIHR Biomedical Research Centre, London, UK; 4 Centre of Applied Statistics Courses, University College London, London, UK; 5 Wellcome/ EPSRC Centre for Interventional and Surgical Sciences, London, UK; 6 Clinical Radiology, St George's Healthcare NHS Trust, London, UK; 7 Clinical Radiology, Kings College Hospital NHS Foundation Trust, London, UK; 8 Clinical Radiology, Royal Wolverhampton Hospitals NHS Trust, Wolverhampton, UK; 9 School of Allied Health Professions, Faculty of Medicine, Health and Social Care, Canterbury Christ Church University, Canterbury, UK; 10 Clinical Radiology, University College London Hospitals NHS Foundation Trust, London, UK; 11 Oxford University Hospitals NHS Foundation Trust, Oxford, UK; 12 Emergency Medicine Research Oxford, Oxford University Hospitals NHS Foundation Trust, Oxford, UK; 13 Orthopaedic Surgery, Sandwell and West Birmingham Hospitals NHS Trust, Birmingham, UK

**Keywords:** Diagnostic Imaging, Paediatric orthopaedics, ACCIDENT & EMERGENCY MEDICINE

## Abstract

**Introduction:**

Paediatric fractures are common but can be easily missed on radiography leading to potentially serious implications including long-term pain, disability and missed opportunities for safeguarding in cases of inflicted injury. Artificial intelligence (AI) tools to assist fracture detection in adult patients exist, although their efficacy in children is less well known. This study aims to evaluate whether a commercially available AI tool (certified for paediatric use) improves healthcare professionals (HCPs) detection of fractures, and how this may impact patient care in a retrospective simulated study design.

**Methods and analysis:**

Using a multicentric dataset of 500 paediatric radiographs across four body parts, the diagnostic performance of HCPs will be evaluated across two stages—first without, followed by with the assistance of an AI tool (BoneView, Gleamer) after an interval 4-week washout period. The dataset will contain a mixture of normal and abnormal cases. HCPs will be recruited across radiology, orthopaedics and emergency medicine. We will aim for 40 readers, with ~14 in each subspecialty, half being experienced consultants. For each radiograph HCPs will evaluate presence of a fracture, their confidence level and a suitable simulated management plan. Diagnostic accuracy will be judged against a consensus interpretation by an expert panel of two paediatric radiologists (ground truth). Multilevel logistic modelling techniques will analyse and report diagnostic accuracy outcome measures for fracture detection. Descriptive statistics will evaluate changes in simulated patient management.

**Ethics and dissemination:**

This study was granted approval by National Health Service Health Research Authority and Health and Care Research Wales (REC Reference: 22/PR/0334). IRAS Project ID is 274 278. Funding has been provided by the National Institute for Heath and Care Research (NIHR) (Grant ID: NIHR-301322). Findings from this study will be disseminated through peer-reviewed publications, conferences and non-peer-reviewed media and social media outlets.https://www.isrctn.com/ISRCTN12921105

**Trial registration number:**

ISRCTN12921105.

**STRENGTHS AND LIMITATIONS OF THIS STUDY**

- Performing a large multireader study evaluating paediatric fracture detection with and without artificial intelligence (AI) assistance across different medical subspecialties and experience levels will better evaluate which healthcare professionals benefit most from AI assistance.
- Our imaging dataset represents a range of children’s ages and body parts across multiple National Health Service trusts to comprehensively evaluate performance of a commercially available AI algorithm.
- The UK-based population used in this study, lack of patient history with predefined simulated clinical management choices may not exactly mimic real-world practices and outcomes.
- Replicating societal and ethical biases, with a comprehensive health economic evaluation of providing AI assistance for fracture detection is difficult to achieve, but our study will provide a guide for future studies.

## Introduction

 Approximately, half of all the 12 million children (<16 years) in the UK will fracture a bone during childhood.^1 2^ Radiographic imaging is the first-line imaging tool for assessing the presence and extent of injury. Unfortunately, the interpretation of paediatric fractures is challenging for many healthcare professionals (HCPs) as children sustain different types of injury to adults, which can sometimes be subtle to identify (eg, buckle or Salter Harris fractures), exhibit a wide range of normal appearances across different ages and, sometimes normal physes can be mistaken for injuries. Furthermore, the level of experience of HCPs varies and while patients should, under best practice principles, not be discharged from hospital without a radiology report, in reality, this is not usually available.

In one study, researchers found that misdiagnoses were made in 10% of children’s fractures by emergency doctors.^3^,^6^ Unfortunately, due to workforce pressures and staff shortages,^7^ doctors with subspecialist skills in imaging or musculoskeletal injuries are not readily available 24/7 in a busy emergency department. This leads to potential delays in recognising mistakes,^8^,^13^ long-term pain and discomfort for the child and, in some situations, missed opportunities for safeguarding (as fractures can be the first sign of inflicted injury).^14^

Recently, many artificial intelligence (AI) tools have been developed and demonstrated high diagnostic accuracy rates for the detection of fractures on imaging, in some cases to the same or higher accuracy as a radiologist.^15 16^ Many of these tools, however, have been specifically designed for adults, although encouraging results have been demonstrated when these tools have been applied to children.^17 18^ Within the last year, one AI tool has specifically been approved by the US Food and Drug Administration (FDA) for use in children over the age of 2 years. If implemented clinically, it could potentially improve the quality of paediatric care, streamline orthopedic clinic referrals and reduce the likelihood of medical litigation.^19^

Nonetheless, widespread adoption of AI within the National Health Service (NHS) is still nascent, with various barriers to adoption identified, of which lack of sufficient evidence is one major concern.^20 21^ There is, therefore, a vital and crucial need to evaluate how such a tool may help (or hinder) different members of staff in this clinical care pathway and whether the use of such a tool would make any difference to patient management.

### Objectives

In this study, the aim is to evaluate whether using a commercially available AI tool certified for paediatric use could help HCPs make better decisions about patient care.

Primary objective:

- Determine differences in diagnostic accuracy rates of HCPs for paediatric fracture detection, before and after using the AI tool.

Secondary objectives:

- Determine whether differences in accuracy rates or effect of adding AI varies according to job role and experience.
- Determine whether user confidence in fracture diagnosis changes when using the AI tool.
- Determine whether management plans are altered following the use of the AI tool.

## Methods and analysis

### Study design

**Figure 1:**
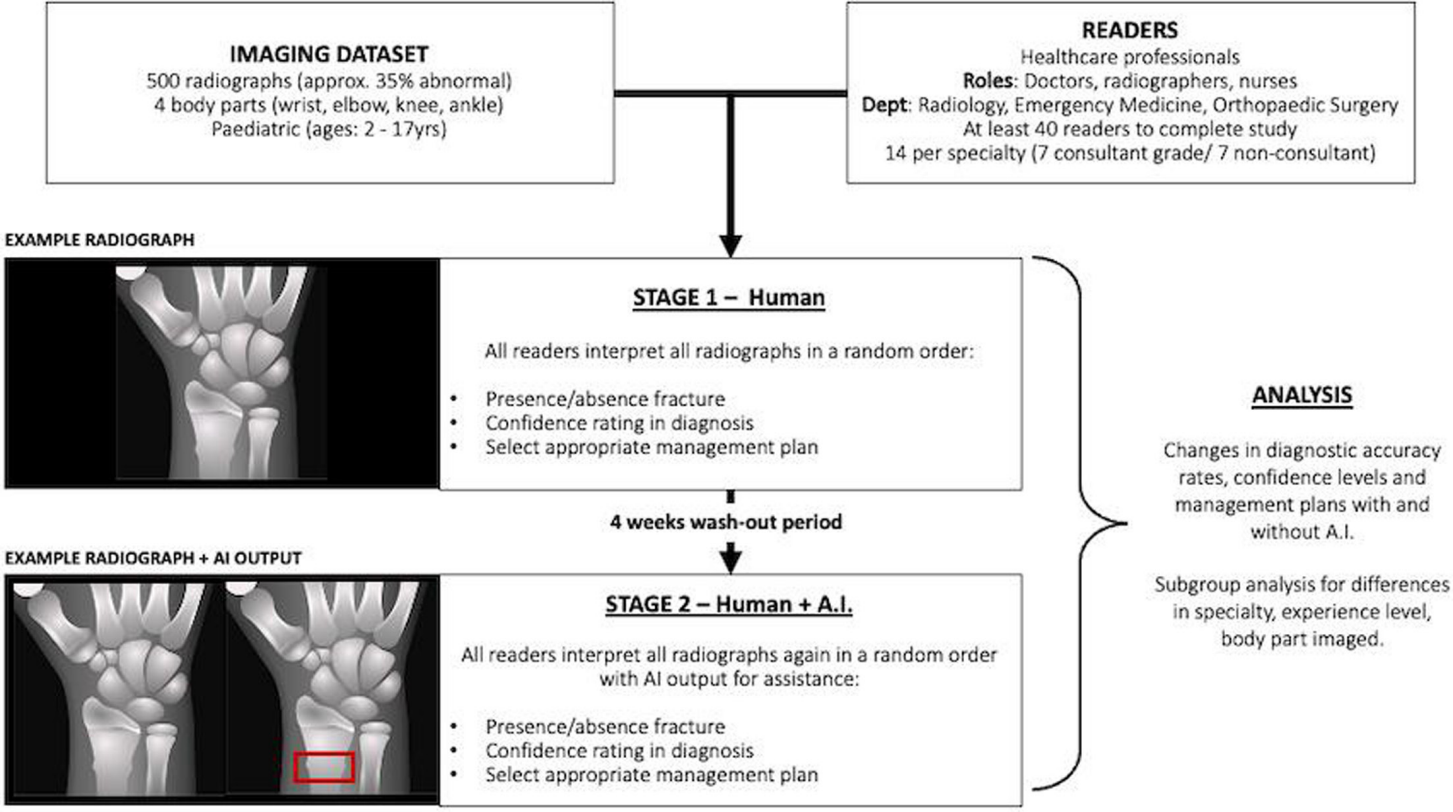
A diagrammatic flow chart of the multireader multicase (MRMC) study outline. AI, artificial intelligence.

The overall study design is of a cross-over multireader multicase (MRMC) study, where each reader (a HCP) will act as their own control across two imaging interpretation stages, with an interval washout period of at least 4 weeks duration (figure 1). Differences in diagnostic accuracy rates between stages, changes in reader’s confidence rates and simulated patient management will be compared. Subgroup analyses will be conducted according to reader specialty area, experience and body part interpreted.

At the first interpretation stage (1 September 2024–31 October 2024), each reader will review a dataset of 500 paediatric limb radiographs (some normal and some abnormal) without AI assistance, then after the washout period (1 November 2024–30 November 2024), they will proceed with the second interpretation stage (1 December 2024–31 January 2025) where they will each read the same dataset with AI assistance. The order of the radiographs, and thus imaged body parts and those with and without abnormalities, will be randomly ordered within the dataset for every reader at each interpretation stage to further reduce recall bias.

Readers will complete the imaging interpretations online, via a password-protected and secure imaging platform (details below). They will be given a 2-month period to complete the exercise and are informed not to seek help in the imaging interpretation with anyone else. Clinical information associated with each radiograph will include the age of patient and gender. Mechanism of injury, pain location, history will not be provided. The reader will be asked to assume that there is generalised pain at the joint in question, and no significant medical history (ie, no genetic or metabolic bone disorder or known malignancy).

**Figure 2:**
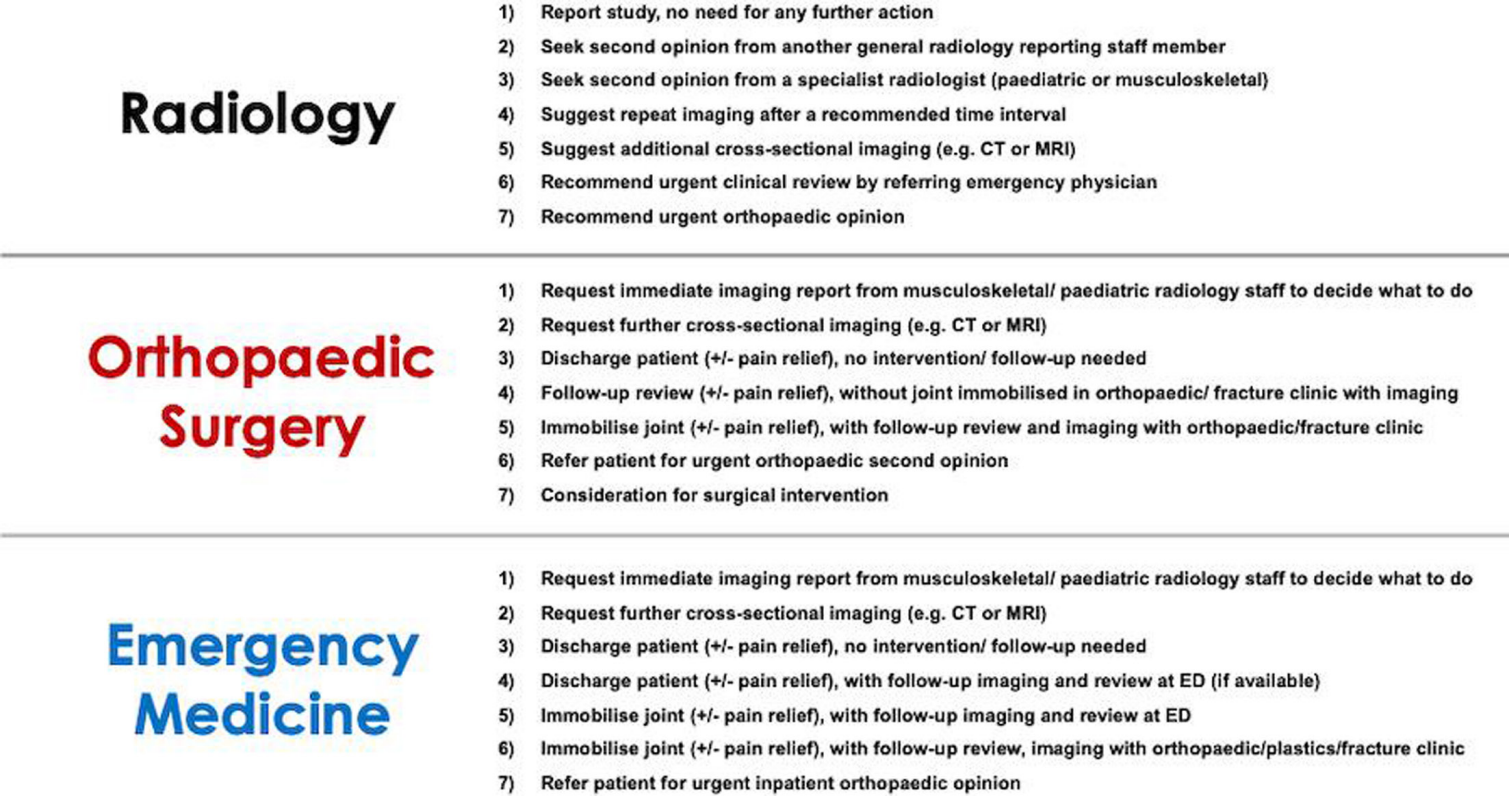
Simulated patient management options provided to different subspecialty readers recruited in this study. For every case reviewed, the reader will be asked to select the single best treatment plan. They will be presented with slightly different options depending on their area of medical expertise. ED, emergency department.

Feedback requested from each reader for each radiograph will include:

1. Marking the site of a fracture on each image (or selecting ‘no fracture’).
2. Confidence in their decision using a 5-point Likert scale (1=not confident; 5=absolutely certain).
3. Selecting the most likely management for the patient. This will be done by providing each reader with a drop-down menu of seven predefined plans, tailored to each subspecialty, with the option for free-text comment. Examples of the different options are provided in figure 2.

### Inclusion and exclusion criteria (imaging cases)

500 anonymised paediatric radiographic examinations (‘cases’) will be derived from a larger 5-year retrospective dataset of appendicular radiographs acquired in children from two NHS trusts (King’s College Hospital NHS Foundation Trust and St George’s University Hospitals NHS Foundation Trust). Both trusts are major trauma centres located in London, UK serving adult and paediatric cases. All radiographic imaging was acquired as part of routine clinical care. No change in patient management will occur as a result of this study, nor was any change in the usual imaging protocol required for this retrospective data collection.

We will include a mixture of normal and abnormal radiographic examinations according to the minimum ratio determined by our sample size calculation below. Four body parts will be used: wrist, elbow, knee and ankle. These were chosen because limbs account for 81.5% of all paediatric injuries,^22^ with those occurring at the knee, wrist and elbow being most commonly missed.^23^ Wrist and elbow fractures encompass 20.5% of all paediatric fractures and missed fractures in these areas are one of the most common reasons for litigation in children’s orthopaedic care.^24^ Although ankle fractures are less common, they account for up to 25% of all growth plate injuries^25^ and thus carry a high potential for long-term growth disorders if misdiagnosed.

Our inclusion criteria for all radiographic imaging in the subset of 500 cases include:

- Children aged between 2 and 18 years old (as the intended commercial AI tool is not regulated for children under 2 years of age).

In order to effectively evaluate how well the AI tool could help HCPs identify easily missed fractures, we will include abnormal radiographs that do not contain ‘obvious’ fractures. Obvious fractures will be defined as any fracture that meets at least one of the following criteria and therefore excluded:

- Any imaging with a ‘Red Dot’ annotation on the radiograph that cannot be removed (denoting the presence of either a true or false abnormality identified by the radiographer).
- Any fracture which is angulated by more than 5°.
- Any fracture which is displaced (>5 mm) or comminuted (multifragmented).
- Any fracture impacted/shortened by >5 mm.
- Any fracture with obvious callus formation/sclerosis.

As we will be evaluating the performance of acute fracture detection in an emergency setting, we will exclude healing fractures. Incidental bone lesions will also be excluded from our study, however, normal anatomical variants will be included.

### Study cohort characteristics (imaging cases)

Cohort demographic characteristics for whole population and abnormal dataset for this study are available in online supplemental tables 1–3, with demographic data on normal dataset in online supplemental table 4. Overall, 500 different radiographic examinations across 500 different paediatric patients (none included more than once) will be used (comprising 183 fractures in 181 patients). The dataset will consist of 256 boys (97 with fractures) and 245 girls (84 with fractures), with mean age of 10 years (range 2–17 years).

The most common fractured bone in our dataset is the distal radius (41/181, 22.4% fractures), and the three the most common fracture types are Salter Harris 2 type injury (41/183, 22.4%), buckle fracture (39/183, 21.3%) and transverse fracture (28/183, 15.3%).

We will invite readers from different specialties and experience levels to participate. These will include HCPs working in radiology (including doctors and reporting radiographers), the emergency medicine department (including doctors and senior triage nurses) and orthopaedic surgeons. All experience levels will be welcome to participate, including those with a subspecialty interest within their field (eg, paediatric orthopaedic surgery, paediatric radiology). Readers will, however, be excluded if they do not routinely review paediatric radiographs as part of their expected job role.

Radiologists and reporting radiographers will be recruited voluntarily through society newsletter announcements via the European Society of Paediatric Radiology (ESPR), European Society of Skeletal Radiology (ESSR) and the British Societies of Paediatric Radiology and Skeletal Radiology (BSPR, BSSR) as well as Society of Radiographers (SoR). Emergency medical staff will be recruited through existing local collaborations, via the Royal College of Emergency Medicine (RCEM) and Association of Paediatric Emergency Medicine (APEM). Orthopaedic surgical colleagues will be recruited also via local collaborations, and via the British Society for Children’s Orthopaedic Surgery (BSCOS). All announcements and invitations to participate will be also posted via study collaborators through their personal social media channels.

We anticipate a minimum of 40 readers (approximately 14 readers from each specialty) equally split between trainees and consultant-level experience. Each reader will be provided instructions on how to participate in the study and complete a consent form online asking about their demographic details, job role/specialty and experience level. Prior to any interpretation, a short video and instruction sheet on how to use the online reporting platform will be provided. Reporters will be asked to replicate their usual reporting practices as far as possible (eg, use of a suitable monitor, dim lighting) and would be able to use reference tools (eg, textbooks or websites) that they would normally consult for this task, but just not to consult other reporters.

### Intervention (AI tool)

Our ‘intervention’ will be the use of a commercially available AI tool called ‘BoneView’ (V.2.3.0) produced by a French AI vendor called Gleamer (Paris, France. https://www.gleamer.ai/). The tool received conformity (CE Class 2A EU MDR and FDA) approval for fracture detection in adults and children (aged >2 years old) in March 2022^26^ on full-resolution Digital Imaging and Communications in Medicine (DICOM) images. This product was chosen as it was the first to achieve FDA approval for use in children and had the greatest evidence basis among all commercial products for fracture detection on radiographs.^19^ The full details of how the deep learning algorithm was developed and tested have been described in the existing literature,^17 27 28^ therefore, only a brief overview of how the product was developed is provided here.

The algorithm is a Deep Convolutional Neural Network-based on the object detection framework ‘Detectron 2’ written and further engineered in Pytorch (V.1.3). It was developed based on a data set of 312 602 radiographs from patients across over 60 radiology departments collected from January 2011 to May 2021. 30% of the radiographs included in the dataset were paediatric (<21 years). When the algorithm confidence surpasses that of a predefined threshold set during algorithm development, the AI tool (BoneView) will create an output of a duplicate radiograph in the imaging examination with either a region of interest on the radiograph with a white square box stating presence of a fracture, a region of interest on the radiograph with a dashed white square box stating ‘indeterminate’ fracture or no overlay with a note below the image stating no fracture. The data set of the present study does not overlap with any examinations used in the development dataset used to create the AI tool, and nor with any data in this study be used to further train the commercial AI tool.

There have been at least three prior publications evaluating the performance of this AI tool within a French, American and Swiss paediatric dataset across a similar range of body parts proposed in this protocol.^17 18 29^ Two of these studies have included a smaller dataset than this planned study (n=300)^17 18^ with an equal split of normal and abnormal cases (not reflective of clinical practice). One was a MRMC study design using eight radiologist readers.^18^ None of the prior studies included a simulated patient management plan component, nor a multidisciplinary team of readers as this study is planning to.

Gleamer has provided its AI tool free of charge for evaluation in this trial but has no involvement in the study design, data analysis, reader recruitment or the decision to publish the final results.

### Reference (ground truth) standard

We will use a consensus interpretation by an expert panel of two paediatric radiologists, both with at least 5 years of subspecialist radiology experience as the reference standard (so-called ‘ground truth’) for this dataset. A bounding box around the entire area of bone fracture on each image (if present) will be assigned where the examination is abnormal. Reference radiologists will have access to the radiographic imaging and original imaging report when setting the ground truth bounding box, as well as any follow-up imaging available; none of this will be available to recruited readers. Disagreements will be resolved by a third musculoskeletal radiologist (with similar experience level).

### Data deidentification and secure storage

Scans selected for the study will be deidentified using a software called XNAT V.3.2.4,^30^ an open source research platform for image-based biomedical research, before being uploaded to a secure image viewing platform for reader interpretation. Access to the platform will be controlled via separate user accounts and passwords for each recruited reader.

All study data generated by the readers’ interpretations will be entered into a password-protected and secure database. Individual reader accuracy scores will be anonymised, and the research team will not have access to the identifying link between the participants’ personal details and the data. Data about the readers’ experience level and subspecialty will be retained to allow group comparisons.

All research staff will comply with the requirements of the Data Protection Act 2018^31^ with regard to the collection, storage, processing and disclosure of personal information and will uphold the Act’s core principles. Data will be collected and maintained according to Good Clinical Practice standards.^32^

### Statistical methods/data analysis plan

The STARD-AI (Standards for Reporting of Diagnostic Accuracy - Artificial Intelligence) and CLAIM (Checklist for AI in Medical Imaging) guidelines will be adhered to in the reporting of this study.^33 34^ Diagnostic accuracy of the readers (with and without AI assistance) for each body part will be derived (ie, sensitivity, specificity, positive predictive value and negative predictive value). A true positive result will be counted if a mark made by a reader at the site of a suspected fracture falls within the area of the predefined ‘ground truth bounding box’ area set by the reference radiologists. If the mark made by a reader falls outside a bounding box, it will be counted as a false positive. Where a bounding box was set by the ground truth, but no mark made on the image then a false negative result will be assigned to the reader.

Estimates of these accuracy statistics will be derived through multilevel logistic regression models, with the reader included as a random-intercept. From these models, we will report 95% CIs, and p values (significance level set at 5%). Independent variables will be added, including the reader’s job role and experience, to assess their relationship with diagnostic accuracy. We will additionally present these same statistics to the reader, as derived through the random effects of the models, to explore the relationship between sensitivity and specificity. Intraobserver variability for the diagnostic accuracy of fracture detection before and after the use of AI assistance will also be evaluated, with subanalysis conducted to account for different reader medical specialty subgroups.

We will assess for differences in confidence scores for correctly identified images between HCPs with and without AI guidance, and also whether there were significant differences between readers across specialties and experience levels. We will also evaluate how an indeterminate AI reading affects reader decisions.

Changes in clinical management will be compared using descriptive statistics (ie, frequency and percentages) to determine, for example, how many children would be discharged with a missed fracture, or unnecessary second opinions/additional imaging sought for cases with and without AI assistance. This could provide information to help estimate potential future benefit and cost savings to the NHS at a future clinical implementation stage, where appropriate.

### Sample size and power calculation

Using the sample size tables published by Obuchowski *et al* for ‘Receiver Operating Characteristic Studies’,^35^ the study has been powered to detect small differences in the AUC (area under the receiver operating curve) of 0.05, with power of 80% and type 1 error rate of 5% between reader and AI algorithm performance. Assuming that the dataset will be representative of clinical practice with at least 20% abnormal cases, our sample size would need to be at least 112 examinations for at least 10 readers, per body part.

In order to ensure better representation of different abnormal findings, we have increased the number of examinations to 125 per body part, with approximately one-third of the cases being abnormal (ie, between 44 and 46 abnormal cases per body part, see online supplemental tables 1–3).

### Patient and public involvement

In designing this research protocol and in the application for the funding, the NIHR GOSH Biomedical Research Centre Patient and Public Advisory Groups for research were consulted which included ‘The Young Persons Advisory Group (YPAG)’ (comprising 24 young people, aged 11–21 years) and ‘The Parent and Carer Advisory Group (PCAG)’ (comprising 5 parent representatives).^36^ Many of the children were familiar with the concept of AI,^37^ and of these, four YPAG and three PCAG members volunteered to form the ‘FRACTURE Patient and Public Involvement & Engagement (PPIE) Steering Committee’ for this project and related works.^38^ Their input has confirmed to us that patients prefer to see how doctors can be helped (rather than replaced) by AI, and therefore, this study aims to understand if AI can enhance current clinical practices and the impact this could have on patient care.

### Ethics and dissemination

#### Human research ethics committee approval

This study was granted approval by NHS Health Research Authority (HRA) and Health and Care Research Wales (HCRW) (REC Reference: 22/PR/0334). IRAS Project ID is 274 278. Informed consent was not required for the use of fully anonymised, retrospective imaging data for this study. Written consent will be received by all readers within this study prior to the interpretation exercises.

### Intended publications and research dissemination

Datasets generated and/or analysed during the current study are not publicly available due to data confidentiality agreements with data custodians. Results generated by the research will be made publicly available at the summary level. Manuscripts addressing the study aims will be published in peer-reviewed journals and will also be presented at relevant national and international conferences. Findings will also be disseminated via social media and online blogs.

Study outcomes will be disseminated to all relevant clinical and non-clinical stakeholders which include our FRACTURE PPIE Steering Group, the wider Great Ormond Street Hospital YPAG and PCAG members, members of the ESPR, ESSR, BSPR, BSSR, SoR, RCEM, APEM, BSCOS, members of the NIHR Imaging Science Working Group and also the Clinical AI interest group of the Alan Turing Institute. The findings and awareness raised by the study and its dissemination will help inform future AI evaluation for paediatric healthcare, policy decisions and raise awareness of AI training needs for various multidisciplinary subspecialties and HCPs who may encounter such tools as part of their role.

## supplementary material

10.1136/bmjopen-2024-084448 online supplemental file 1

## References

[1] Jones IE, Williams SM, Dow N (2002). How many children remain fracture-free during growth? a longitudinal study of children and adolescents participating in the Dunedin Multidisciplinary Health and Development Study. Osteoporos Int.

[2] Cooper C, Dennison EM, Leufkens HG (2004). Epidemiology of Childhood Fractures in Britain: A Study Using the General Practice Research Database. J Bone Miner Res.

[3] Eakins C, Ellis WD, Pruthi S (2012). Second opinion interpretations by specialty radiologists at a pediatric hospital: rate of disagreement and clinical implications. AJR Am J Roentgenol.

[4] Taves J, Skitch S, Valani R (2018). Determining the clinical significance of errors in pediatric radiograph interpretation between emergency physicians and radiologists. CJEM.

[5] Klein EJ, Koenig M, Diekema DS (1999). Discordant radiograph interpretation between emergency physicians and radiologists in a pediatric emergency department. Pediatr Emerg Care.

[6] Al-Sani F, Prasad S, Panwar J (2020). Adverse Events from Emergency Physician Pediatric Extremity Radiograph Interpretations: A Prospective Cohort Study. *Acad Emerg Med*.

[7] Royal College of Radiologists R (2022). RCR Clinical Radiology Workforce Census.

[8] Halliday K, Drinkwater K, Howlett DC (2016). Evaluation of paediatric radiology services in hospitals in the UK. Clin Radiol.

[9] McColgan M, Winch R, Clark SJ (2017). The changing UK paediatric consultant workforce: report from the Royal College of Paediatrics and Child Health. Arch Dis Child.

[10] Aquino MR, Maresky HS, Amirabadi A (2020). After-hours radiology coverage in children’s hospitals: a multi-center survey. Pediatr Radiol.

[11] Davies FC, Newton T (2015). Paediatric emergency medicine consultant provision in the UK: are we there yet?. Arch Dis Child.

[12] Radiologists RCo (2015). National Audit of Paediatric Radiology Services in Hospitals.

[13] Commission CCQ Radiology review: A national review of radiology reporting within the NHS in England 2018.

[14] Karmazyn B, Wanner MR, Marine MB (2019). The added value of a second read by pediatric radiologists for outside skeletal surveys. Pediatr Radiol.

[15] Kuo RYL, Harrison C, Curran T-A (2022). Artificial Intelligence in Fracture Detection: A Systematic Review and Meta-Analysis. Radiology.

[16] Shelmerdine SC (2020). Artificial intelligence for fracture detection and classification in paediatric radiology: a systematic review prospero international prospective register of systematic.

[17] Hayashi D, Kompel AJ, Ventre J (2022). Automated detection of acute appendicular skeletal fractures in pediatric patients using deep learning. Skeletal Radiol.

[18] Nguyen T, Maarek R, Hermann A-L (2022). Assessment of an artificial intelligence aid for the detection of appendicular skeletal fractures in children and young adults by senior and junior radiologists. Pediatr Radiol.

[19] Pauling C, Kanber B, Arthurs OJ (2023). Commercially available artificial intelligence tools for fracture detection: the evidence. BJROpen.

[20] Eltawil FA, Atalla M, Boulos E (2023). Analyzing Barriers and Enablers for the Acceptance of Artificial Intelligence Innovations into Radiology Practice: A Scoping Review. Tomography.

[21] Royal College of Radiologists R Overcoming Barriers to AI Implementation in Imaging: Outcome of an RCR Expert Stakeholder Day 2022.

[22] Jones S, Tyson S, Young M (2019). Patterns of moderate and severe injury in children after the introduction of major trauma networks. Arch Dis Child.

[23] Molins CM, Martinez M (2016). Common Missed Radiographic Findings. Emerg Med Rep.

[24] Atrey A, Nicolaou N, Katchburian M (2010). A review of reported litigation against English health trusts for the treatment of children in orthopaedics: present trends and suggestions to reduce mistakes. *J Child Orthop*.

[25] Crawford AH, Al-Sayyad MJ (2003). Fractures and Dislocations of the Foot and Ankle.

[26] FDA Gleamer BoneView v2.5.0 FDA documentation Ref.

[27] Guermazi A, Tannoury C, Kompel AJ (2022). Improving Radiographic Fracture Recognition Performance and Efficiency Using Artificial Intelligence. Radiology.

[28] Duron L, Ducarouge A, Gillibert A (2021). Assessment of an AI Aid in Detection of Adult Appendicular Skeletal Fractures by Emergency Physicians and Radiologists: A Multicenter Cross-sectional Diagnostic Study. Radiology.

[29] Altmann-Schneider I, Kellenberger CJ, Pistorius S-M (2024). Artificial intelligence-based detection of paediatric appendicular skeletal fractures: performance and limitations for common fracture types and locations. Pediatr Radiol.

[30] Marcus DS, Olsen TR, Ramaratnam M (2007). The Extensible Neuroimaging Archive Toolkit: an informatics platform for managing, exploring, and sharing neuroimaging data. Neuroinformatics.

[31] Gov uk (2018). The Data Protection Act.

[32] Authority NHR (2020). Good Clinical Practice.

[33] Mongan J, Moy L, Kahn CE (2020). Checklist for Artificial Intelligence in Medical Imaging (CLAIM): A Guide for Authors and Reviewers. *Radiol Artif Intell*.

[34] Sounderajah V, Ashrafian H, Aggarwal R (2020). Developing specific reporting guidelines for diagnostic accuracy studies assessing AI interventions: The STARD-AI Steering Group. Nat Med.

[35] Obuchowski NA (2000). Sample size tables for receiver operating characteristic studies. AJR Am J Roentgenol.

[36] GenerationR Generation R - Young People Improving Research 2023.

[37] Visram S, Leyden D, Annesley O (2023). Engaging children and young people on the potential role of artificial intelligence in medicine. Pediatr Res.

[38] Study F (2020). FRACTURE study website. https://fracturestudy.com/.

