# Supplementary material for: Artificial intelligence (AI) for paediatric fracture detection: a multireader multicase (MRMC) study protocol
Source: BMJ Open. 2024 Dec 7;14(12):e084448. doi: 10.1136/bmjopen-2024-084448 (PMC11628946; doi:10.1136/bmjopen-2024-084448)
Supplement: online supplemental file 1 [file bmjopen-14-12-s001.pdf]

**SUPPLEMENTARY MATERIAL**

**Table S1 - Complete imaging dataset demographics**

Demographic characteristics for all cases (both normal and abnormal) by body part and NHS trust (n = 500).

KCH – King's College Hospital NHS Foundation Trust; SGH – St. George's University Hospitals NHS Foundation Trust

| | **Laterality** | | **Gender** | | **Total Cases** | **Age Range (years)** | **Median Age (years)** | **Average Age (years)** |
|---|---|---|---|---|---|---|---|---|
| | **R** | **L** | **M** | **F** | | | | |
| **Wrist** | | | | | | | | |
| KCH | 26 | 33 | 24 | 35 | 59 | 2 – 15 | 11 | 10 |
| SGH | 32 | 34 | 40 | 26 | 66 | 4 – 16 | 11 | 12 |
| Combined Wrist | 58 | 67 | 64 | 61 | 125 | 2 – 16 | 11 | 11 |
| **Elbow** | | | | | | | | |
| KCH | 26 | 31 | 26 | 31 | 57 | 2 – 14 | 7 | 7 |
| SGH | 35 | 33 | 37 | 31 | 68 | 2 – 14 | 8 | 8 |
| Combined Elbow | 61 | 64 | 63 | 62 | 125 | 2 – 14 | 8 | 8 |
| **Knee** | | | | | | | | |
| KCH | 34 | 26 | 29 | 31 | 60 | 2 – 15 | 10 | 10 |
| SGH | 34 | 31 | 37 | 28 | 65 | 3 – 16 | 11 | 11 |
| Combined Knee | 68 | 57 | 66 | 59 | 125 | 2 – 17 | 11 | 10 |
| **Ankle** | | | | | | | | |
| KCH | 25 | 27 | 27 | 27 | 52 | 3 – 14 | 10 | 10 |
| SGH | 39 | 34 | 36 | 36 | 73 | 3 – 16 | 10 | 10 |
| Combined Ankle | 64 | 61 | 63 | 63 | 125 | 3 – 16 | 10 | 10 |
| **TOTAL CASES** | **244 (48.8%)** | **257 (51.4%)** | **256 (51.2%)** | **245 (49.0%)** | **500 (100%)** | **2 – 17** | **10** | **10** |

**Table S2 - Abnormal imaging dataset demographics**
Demographic characteristics for abnormal radiographic examinations, separated by body part and NHS trust (n = 181).
KCH – King's College Hospital NHS Foundation Trust; SGH – St. George's University Hospitals NHS Foundation Trust

| | Laterality | | Gender | | Total Examinations | Age Range (years) | Median Age (years) | Average Age (years) |
|---|---|---|---|---|---|---|---|---|
| | R | L | M | F | | | | |
| **Wrist** | | | | | | | | |
| KCH | 12 | 13 | 11 | 14 | 25 | 3 – 15 | 10 | 10 |
| SGH | 11 | 9 | 14 | 6 | 20 | 8 – 16 | 12 | 12 |
| Combined Wrist | 23 | 22 | 25 | 20 | 45 | 3 – 16 | 11 | 11 |
| **Elbow** | | | | | | | | |
| KCH | 11 | 12 | 12 | 11 | 23 | 3 – 14 | 5 | 6 |
| SGH | 14 | 9 | 13 | 10 | 23 | 3 – 14 | 6 | 6 |
| Combined Elbow | 25 | 21 | 25 | 21 | 46 | 3 – 14 | 6 | 6 |
| **Knee** | | | | | | | | |
| KCH | 13 | 9 | 9 | 13 | 22 | 3 – 15 | 10 | 9 |
| SGH | 12 | 12 | 18 | 6 | 24 | 3 – 16 | 11 | 11 |
| Combined Knee | 25 | 21 | 27 | 19 | 46 | 3 - 17 | 10 | 10 |
| **Ankle** | | | | | | | | |
| KCH | 6 | 8 | 6 | 8 | 14 | 3 – 13 | 10 | 9 |
| SGH | 17 | 13 | 14 | 16 | 30 | 3 – 16 | 10 | 9 |
| Combined Ankle | 23 | 21 | 20 | 24 | 44 | 3 – 16 | 10 | 9 |
| **TOTAL CASES** | **96 (53.0%)** | **85 (47.0%)** | **97 (53.5%)** | **84 (46.4%)** | **181 (100%)** | **3 - 17** | **9** | **9** |

**Table S3 - Abnormal imaging dataset pathologies**
Abnormal fracture pathologies in our imaging dataset by fracture type and body part (n, %).

| | **Buckle** | **Bowing** | **Salter Harris 1** | **Salter Harris 2** | **Salter Harris 3** | **Salter Harris 4** | **Greenst ick** | **Avulsio n** | **Transve rse** | **Spiral** | **Suprac ondylar** | **Epiphys eal** | **Vertical** | **TOTAL** |
|---|---|---|---|---|---|---|---|---|---|---|---|---|---|---|
| **WRIST** | | | | | | | | | | | | | | |
| Distal radius | 25 | 1 | 1 | 8 | 1 | 0 | 3 | 0 | 2 | 0 | 0 | 0 | 0 | **41 (22.4)** |
| Distal ulna | 0 | 0 | 0 | 0 | 0 | 0 | 0 | 0 | 3 | 0 | 0 | 0 | 0 | **3 (1.6)** |
| Metacarpus | 0 | 0 | 0 | 0 | 0 | 0 | 0 | 0 | 2 | 0 | 0 | 0 | 0 | **2 (1.1)** |
| **ELBOW** | | | | | | | | | | | | | | |
| Proximal radius | 1 | 0 | 0 | 4 | 0 | 0 | 0 | 1 | 6 | 0 | 0 | 0 | 0 | **12 (6.6)** |
| Proximal ulna | 0 | 0 | 0 | 0 | 0 | 0 | 0 | 0 | 5 | 0 | 0 | 0 | 0 | **5 (2.7)** |
| Distal humerus | 0 | 0 | 0 | 0 | 0 | 0 | 0 | 2 | 1 | 0 | 26 | 0 | 0 | **29 (15.8)** |
| **KNEE** | | | | | | | | | | | | | | |
| Distal femur | 2 | 0 | 0 | 3 | 1 | 0 | 0 | 1 | 2 | 0 | 0 | 0 | 0 | **9 (4.9)** |
| Proximal tibia | 6 | 0 | 0 | 5 | 0 | 1 | 0 | 8 | 0 | 0 | 0 | 0 | 0 | **23 (12.6)** |
| Proximal fibula | 0 | 0 | 0 | 0 | 0 | 0 | 0 | 0 | 1 | 0 | 0 | 0 | 0 | **2 (1.1)** |
| Patella | 0 | 0 | 0 | 0 | 0 | 0 | 0 | 6 | 6 | 0 | 0 | 0 | 1 | **13 (7.1)** |
| **ANKLE** | | | | | | | | | | | | | | |
| Talus | 0 | 0 | 0 | 0 | 0 | 0 | 0 | 1 | 0 | 0 | 0 | 0 | 0 | **1 (0.5)** |
| Distal fibula | 5 | 0 | 1 | 12 | 0 | 0 | 0 | 2 | 0 | 0 | 0 | 0 | 0 | **26 (14.2)** |
| Distal tibia | 0 | 0 | 0 | 9 | 2 | 1 | 0 | 0 | 0 | 5 | 0 | 0 | 0 | **17 (9.3)** |
| **TOTAL CASES** | **39 (21.3)** | **1 (0.5)** | **2 (1.1)** | **41 (22.4)** | **4 (2.2)** | **2 (1.1)** | **3 (1.6)** | **21 (11.5)** | **28 (15.3)** | **5 (2.7)** | **26 (14.2%)** | **10 (5.5)** | **1 (0.5)** | **183** |

**Table S4 - Normal imaging dataset demographics**

Imaging dataset – demographic characteristics for normal cases by body part and NHS trust (n = 319)

KCH – King's College Hospital NHS Foundation Trust; SGH – St. George's University Hospitals NHS Foundation Trust

| | Laterality | | Sex | | Total Cases | Age Range (years) | Median Age (years) | Average Age (years) |
|---|---|---|---|---|---|---|---|---|
| | R | L | M | F | | | | |
| **Wrist** | | | | | | | | |
| KCH | 14 | 20 | 13 | 21 | 34 | 2-15 | 11 | 10 |
| SGH | 21 | 25 | 26 | 20 | 46 | 4-16 | 10 | 11 |
| Combined Wrist | 35 | 45 | 39 | 41 | 80 | 2-16 | 11 | 11 |
| **Elbow** | | | | | | | | |
| KCH | 15 | 19 | 14 | 20 | 34 | 2-13 | 8 | 8 |
| SGH | 21 | 24 | 24 | 21 | 45 | 2-14 | 10 | 9 |
| Combined Elbow | 36 | 43 | 38 | 41 | 79 | 2-14 | 9 | 9 |
| **Knee** | | | | | | | | |
| KCH | 17 | 21 | 20 | 18 | 38 | 2-15 | 10 | 10 |
| SGH | 19 | 22 | 19 | 22 | 41 | 3-16 | 11 | 10 |
| Combined Knee | 36 | 43 | 39 | 40 | 79 | 2-16 | 11 | 10 |
| **Ankle** | | | | | | | | |
| KCH | 19 | 19 | 19 | 19 | 38 | 4-14 | 10 | 10 |
| SGH | 22 | 21 | 23 | 20 | 43 | 3-16 | 10 | 10 |
| Combined Ankle | 41 | 40 | 42 | 39 | 81 | 3-16 | 10 | 10 |
| **TOTAL CASES** | **148 (46.4%)** | **171 (53.9%)** | **158 (49.8%)** | **161 (50.5%)** | **319 (100%)** | **2 - 16** | **10** | **10** |
