# Supplementary material for: Artificial intelligence (AI) for paediatric fracture detection: a multireader multicase (MRMC) study protocol
Source: BMJ Open. 2024 Dec 7;14(12):e084448. doi: 10.1136/bmjopen-2024-084448 (PMC11628946; doi:10.1136/bmjopen-2024-084448)
Supplement: Supplementary file 2 [file bmjopen-2024-084448.reviewer_comments.pdf]

PEER REVIEW HISTORY

BMJ Open publishes all reviews undertaken for accepted manuscripts. Reviewers are asked to complete a checklist review form and are provided with free text boxes to elaborate on their assessment. These free text comments are reproduced below.

ARTICLE DETAILS

Title (Provisional)

Artificial intelligence (AI) for paediatric fracture detection: a multireader multicase (MRMC) study protocol

Authors

Shelmerdine, Susan; Pauling, Cato; Allan, Emma; Langan, Dean; Ashworth, Emily; Yung, Ka-Wai; Barber, Joy; Haque, Saira; Rosewarne, David; Woznitza, Nicholas; Ather, Sarim; Novak, Alex; Theivendran, Kanthan; Arthurs, Owen John

---

VERSION 1 - REVIEW

Reviewer 1

Name McGuirk, Simon

Affiliation Birmingham Women's and Children's Hospitals NHS Foundation Trust, Department of Interventional Radiology

Date 08-Feb-2024

COI None

I read the Study Protocol with interest. It is well written and clearly explains the objectives and potential clinical value of the study. I would be happy to recommend publication.

I have a couple of comments.

Funding Information. Could the order of authors be changed to reflect their order in the authorship?

The potential for intra-observer variation has not been accounted for. This is reportedly up to 20% of cases. Should an assessment of this variation be made?

Will clinical tools used in normal practice be allowed as part of the review process?

For example, can "Keats" be used?

Practice with regard to management of patient is likely to be much more subjective and unit-based. How will this issue be addressed? I am not clear whether there is a "right" answer for this aspect of the study. I appreciate the study will look at a change in management, as much as the type of management recommended.

Obvious fractures will not be included.

I appreciate that the added value of AI may be less, but do the authors preconceptions cloud an assessment of the added value of AI in these cases?

These are idle thoughts for consideration of the team, rather than to necessarily be addressed directly in the paper.

**Reviewer**	**2**
**Name**	**Altmann-Schneider, Irmhild**
**Affiliation**	**University Children's Hospital Zürich, Radiology**
**Date**	**12-Feb-2024**
**COI**	**None**

This is a well-written and complete study protocol. Please find attached a few minor remarks for the authors.

1.

Page 6 line 39-45:

In order to keep the study setting as close as possible to clinical practice, the authors could choose to provide clinical information to the readers, as being aware of the clinical information is one of the advantages of HCPs when compared to AI.

2. The authors acknowledge the assistance on the protocol from members from Gleamer. However, the role of Gleamer in this study is not stated clearly. Does Gleamer provide the software for the study free of charge?

3. Table 3 Fracture distribution

The authors explain in the protocol, that they plan to focus on easily missed fractures and to exclude obvious fractures, which seems a reasonable plan to keep the amount of cases for the readers as low as possible. However, in the final dataset, there are for example only one bowing fracture of the radius and in total 2 SH-I-fractures included. Both fracture types are rare, but also known to be difficult to diagnose confidently for both human readers and AI.

**Reviewer**	**3**
**Name**	**Storey, Helen**
**Affiliation**	**PATH, Diagnostics**
**Date**	**11-Jul-2024**
**COI**	**none**

The authors have prepared a well written manuscript describing an interesting and important study design. A few comments that could be added for greater clarity:

1) In the abstract, add mention of the retrospective nature of the study. It is not feasible to comment on impact to patient care so clarify that outcome, and for all outcomes add the specific endpoints that will be assessed.

2) In the strengths/limitations, the last bullet is maybe less relevant for a retrospective study design. Is there something around generalization of this study population compared to other pediatric groups, maybe nationally or globally?

3) An important consideration with the introduction of AI tools in clinical care is provider perceptions, which can affect if and how tools are used as intended. Will qualitative attributes such as usability and acceptability be evaluated in this study?

4) Add mention that the STARD guidelines will be aligned to in this study reporting.

5) On page 9, line 13, it is noted that the algorithm confidence has a pre-defined threshold that is set during algorithm development. How, if at all, does this change by setting or user or manufacturer lot release?

6) On page 9 line 26, it is noted that 3 other studies have evaluated this tool. What were the findings? Why is this study needed?

7) It would be nice to include a note on how the manufacturer has been engaged in the study, for transparency purposes.

8) Are there any concerns with fatigue asking 40 readers to review 1000 slides each, plus 2 panel reviewers to review 500 slides each, presumably with more attention/effort. Will recommendations/guidelines be given on things like max number per day, number of sequential days?

Thank you for sharing this work for other researchers to learn from.

**Reviewer**	**4**
**Name**	**Kurmis , Andrew**
**Affiliation**	**The University of Adelaide, Medical Specialties**
**Date**	**14-Jul-2024**
**COI**	**N/A**

Likely a very useful planned endeavour, not just for the application to paediatric fractures, but also the safe adoption of AI into clinical practice.

General comments -

1. it is generally considered poor grammatical form in a scientific manuscript to begin a sentence with an abbreviation. This occurs multiple times. Please either use the full form (i.e. non-abbreviated form) or amend such sentences such that this rule is not violated.

2. abbreviations should be introduced sequentially at first use in the main body of the manuscript and then employed consistently thereafter. Please scan through the entire document to ensure this is the case. For example, HCP is introduced L18, P 6, yet the full form is first used L13, P5.

3. the statement L13,P5 - '... patients should not be discharged ...' is too strong an assertion and not universally true. Please amend (perhaps, 'under best practice principles ...'.

4. L25, P10 - wouldn't 'no mark' be recorded as a negative result ? i.e. not a 'false positive' ?

5. L55, P10 - 'et al' is an abbreviation and should be followed by a full stop (i.e. 'et al.').

6. although bio9logically correct, please consider amending 'sex' to 'gender' in the tables and main document. The former term carries different connotations in different parts of the world and the journal's readership audience is wide.

VERSION 1 - AUTHOR RESPONSE

REVIEWER 1:

1. Funding Information. Could the order of authors be changed to reflect their order in the authorship?

   The funding information has been revised to follow the authorship listing

2. The potential for intra-observer variation has not been accounted for. This is reportedly up to 20% of cases. Should an assessment of this variation be made?

   This is an excellent point. We have now included a line within our data analysis to describe how we will evaluate for intra-observer and inter-observer variability within our subhgroup of readers and also all readers overall.

3. Will clinical tools used in normal practice be allowed as part of the review process? For example, can "Keats" be used?

   We allow readers to conduct their reviews in any manner that best replicates their usual clinical practice including access to the internet and reference materials if they would normally have these to hand in the usual clinical setting.

4. Practice with regard to management of patient is likely to be much more subjective and unit-based. How will this issue be addressed? I am not clear whether there is a "right" answer for this aspect of the study. I appreciate the study will look at a change in management, as much as the type of management recommended.

   This is correct, we do not envisage a 'correct' management plan as this will vary according to the local practices and we have not provided any clinical background to the case where we feel it would be easy for the readers to provide a definitive answer to this. We are looking at the simulated changes in practice that might occur should an AI be introduced for this particular use case.

5. Obvious fractures will not be included. I appreciate that the added value of AI may be less, but do the authors preconceptions cloud an assessment of the added value of AI in these cases?

   We agree that by excluding all 'obvious' fractures our dataset will be somewhat biased in nature, and in an ideal world we would like to account for

REVIEWER 2:

6. Page 6 line 39-45: In order to keep the study setting as close as possible to clinical practice, the authors could choose to provide clinical information to the readers, as being aware of the clinical information is one of the advantages of HCPs when compared to AI.

   Thank you for this excellent suggestion. This was our original intention, and we agree that this would allow us to most closely align with clinical practice, however due to limitations on our reader platform and image viewer, it is sadly not possible to provide this additional information. Instead we provide the gender and age of our patient, and provide instructions to our readers that they should imagine that the patient has had some trauma and is complaining of pain in the joint imaged. We agree this is a limitation and added this to the final bullet point of our limitations section.

7. The authors acknowledge the assistance on the protocol from members from Gleamer. However, the role of Gleamer in this study is not stated clearly. Does Gleamer provide the software for the study free of charge?
   Gleamer have provided their AI tool free of charge, but have no role in the protocol design, reader recruitment or influence on the analysis or write-up of the study. We have added this to the methodology section.

8. Table 3 Fracture distribution. The authors explain in the protocol, that they plan to focus on easily missed fractures and to exclude obvious fractures, which seems a reasonable plan to keep the amount of cases for the readers as low as possible. However, in the final dataset, there are for example only one bowing fracture of the radius and in total 2 SH-I-fractures included. Both fracture types are rare, but also known to be difficult to diagnose confidently for both human readers and AI.
   Thank you for this insight. We agree that it would be helpful to include more rare fracture types to better evaluate how we can avoid missing these challenging cases, however despite our large dataset across two trauma centres we were unable to find additional fair examples of these cases to include.

REVIEWER 3:

9. In the abstract, add mention of the retrospective nature of the study. It is not feasible to comment on impact to patient care so clarify that outcome, and for all outcomes add the specific endpoints that will be assessed.
   We have made it now clearer in our abstract that this is a retrospective simulated study. We have clarified the outcome to be diagnostic accuracy for fracture detection.

10. In the strengths/limitations, the last bullet is maybe less relevant for a retrospective study design. Is there something around generalization of this study population compared to other pediatric groups, maybe nationally or globally?

We agree and have now amended the final bullet point to highlight the geographical bias of our patient population.

11. An important consideration with the introduction of AI tools in clinical care is provider perceptions, which can affect if and how tools are used as intended. Will qualitative attributes such as usability and acceptability be evaluated in this study?
We thank the reviewer for this important insight. In fact, although not directly part of this MRMC study, we did ask the readers at recruitment stage to evaluate their own diagnostic accuracy and perceived benefit of AI for other users. We agree this information would be helpful to include in future considerations when implementing AI tools, but do not include this as part of our MRMC trial protocol as we feel it may detract from the image evaluation assessment

12. Add mention that the STARD guidelines will be aligned to in this study reporting.
This has now been included under the 'Data Analysis Plan'

13. On page 9, line 13, it is noted that the algorithm confidence has a pre-defined threshold that is set during algorithm development. How, if at all, does this change by setting or user or manufacturer lot release?
The tool has a predefined threshold that is fixed, and any changes that the manufacturer would like to make will necessitate additional regulatory approval prior to commercial release.

14. On page 9 line 26, it is noted that 3 other studies have evaluated this tool. What were the findings? Why is this study needed?
In the section subheading "Intervention (AI tool)" these three studies are mentioned in the final paragraph. None of these pertain to a UK population and two had a smaller dataset and all had a lower number of readers, none with a multi-disciplinary readership. In other words, our study will be the only one to include a range of healthcare professionals of different disciplines in order to evaluate how different specialties benefit or not from use of AI tool in their

workflow. Furthermore our study will be the only one that includes fractures in a more realistic real world split of normal versus abnormal so it will be more challenging for readers to ‘guess’ an abnormality based on prior knowledge of an equal split of findings.

15. It would be nice to include a note on how the manufacturer has been engaged in the study, for transparency purposes.
Thank you for raising this. We have added this in the subsection relating to the AI tool.

16. Are there any concerns with fatigue asking 40 readers to review 1000 slides each, plus 2 panel reviewers to review 500 slides each, presumably with more attention/effort. Will recommendations/guidelines be given on things like max number per day, number of sequential days?
We agree that reading fatigue can cause for lower quality interpretation and a higher chance of missed findings. We will suggest in our initial instruction email to readers that they do no more than a solid 30 minutes of image interpretation at a time, followed by at least a 10 minute break to pace themselves. Nevertheless, given that all readers are voluntary and likely have set aside specific days / times to help we would like to avoid being too restrictive about how they practice but more than happy to provide general guidance on best practice for optimal wellbeing.

REVIEWER 4:

17. It is generally considered poor grammatical form in a scientific manuscript to begin a sentence with an abbreviation. This occurs multiple times. Please either use the full form (i.e. non-abbreviated form) or amend such sentences such that this rule is not violated.
This has been amended. We have kept the initials of the authorship in the author contributions however even if these are abbreviated, we hope this is allowable.

18. Abbreviations should be introduced sequentially at first use in the main body of the manuscript and then employed consistently thereafter. Please scan through the entire document to ensure this is the case. For example, HCP is introduced L18, P 6, yet the full form is first used L13, P5.

This has been amended.

19. The statement L13,P5 - '... patients should not be discharged ...' is too strong an assertion and not universally true. Please amend (perhaps, 'under best practice principles ...'.

This has been amended.

20. L25, P10 - wouldn't 'no mark' be recorded as a negative result ? i.e. not a 'false positive' ?

Correct, thank you for highlighting this oversight. This would be a false negative result if no mark made when a fracture is present. This has been amended.

21. L55, P10 - 'et al' is an abbreviation and should be followed by a full stop (i.e. 'et al.').

This has been amended.

22. Although biologically correct, please consider amending 'sex' to 'gender' in the tables and main document. The former term carries different connotations in different parts of the world and the journal's readership audience is wide.

This has been amended.

VERSION 2 - REVIEW

**Reviewer**	**2**
**Name**	**Altmann-Schneider, Irmhild**
**Affiliation**	**University Children's Hospital Zürich, Radiology**
**Date**	**24-Sep-2024**

COI

Well-written and complete study protocol. The authors have responded adequately to all comments. No further remarks.

VERSION 2 - AUTHOR RESPONSE

REVIEWER 2:

1. Well-written and complete study protocol. The authors have responded adequately to all comments. No further remarks.

   Thank you for your kind words and time spent re-reviewing our work.
